# Pedicle internal limiting membrane flap technique for very large macular holes: a preliminary report

**DOI:** 10.1186/s40942-020-00248-7

**Published:** 2020-09-21

**Authors:** José Edísio da Silva Tavares Neto, Igor Neves Coelho, Rodrigo Jorge, David Leonardo Cruvinel Isaac, Marcos Pereira de Ávila

**Affiliations:** 1 Department of Ophthalmology, Ribeirão Preto Medical School, University of São Paulo, 3900. Bandeirantes Ave, Ribeirão Preto, SP 14049-900 Brazil; 2 Department of Ophthalmology, Federal University of Goiás, Goiânia, Brazil

**Keywords:** Retina, Macula lutea, Macular hole, Vitreous, Vitrectomy, Inner limiting membrane

## Abstract

**Background:**

Conventional vitrectomy technique for macular hole surgery has a good outcome in small and medium macular holes, but for very large macular holes (minimum linear diameter higher than 700 μm) other techniques were developed aiming to achieve greater rates of closure and visual acuity gain. The purpose of this article is to report the anatomical and functional outcomes of four very large macular hole (MH) cases which have undergone vitrectomy with the pedicle internal limiting membrane (ILM) flap technique.

**Methods:**

This is a retrospective series of four patients with large MH who were treated with vitrectomy and the pedicle ILM flap technique. Comprehensive ophthalmologic evaluation was performed before surgery and included ETDRS best-corrected visual acuity (BCVA) and spectral domain optical coherence tomography (SD-OCT) for MH measures: height, minimum linear diameter (MLD) and external base diameter. The particular detail of this technique is related to ILM flap creation. During the peeling, the ILM was not removed completely from the retina but was left attached to the edges of the macular hole and subsequently trimmed with the vitrectomy probe using the scissors mode.

**Results:**

Four patients with very large MH underwent PPV and the pedicle ILM flap technique was used to pursue macular closure. Median preoperative BCVA was 20/400 (range: 20/320 to 20/400) and median postoperative BCVA was 20/200 (range: 20/320 to 20/200). Of the 4 cases reported, 3 obtained anatomical closure (75%), and also presented BCVA improvement after surgery, considering the last follow-up visit of each case. No additional procedures were performed in either case. One patient demonstrated no anatomic and functional improvement.

**Conclusion:**

The present study describes the first Brazilian case series of very large MH treated by the inverted pedicle ILM flap technique. This technique was associated with anatomic and visual improvement in most cases, and represents an alternative therapeutic approach for large macular holes.

*Trial Registration *Project registered in Plataforma Brasil with CAAE number 30163520.0.0000.5440 and approved in ethics committee   from  Ribeirão Preto Medical School Clinics Hospital, University of São Paulo—Ribeirão Preto, São Paulo, Brazil (appreciation number 3.948.426 gave the approval).

## Background

A macular hole is defined as a full thickness retinal break caused in most cases by vitreoretinal traction [1]. The prevalence of idiopathic macular holes ranges from 0.02% [2] to 0.33% [3] or 0.7% [4], being 4 in 1,000 in the population aged 63–102 years [5]. A study conducted in Minnesota (US) detected an incidence of 7.8 per 100 and per year, with a 3.3/1 female/male ratio [6]. Studies on Asian populations have reported rates of 0.09 and 0.17% [7, 8]. Age of 65 years or older and female sex are the relevant systemic risk factors identified thus far [9, 10]. Bilateral involvement varies considerably from 5 to 16% [11–13].

The role of the vitreous in this disease and its classification started to be understood by Gass with the use of posterior segment biomicroscopy [13–15]. However, it was only with optical coherence tomography that it became possible to better study vitreoretinal traction and macular holes [16]. A good visualization of the vitreoretinal interface permitted the introduction of the concept of vitreomacular adhesion (adhesion with no change in retinal architecture) and vitreomacular traction (adhesion generating anatomical changes), as well as the subdivision of hole size (shortest distance between its margins) into small (< 250 μm), medium (250–400 μm) and large (> 400 μm). This classification also includes information about a primary or secondary etiology (high myopia, trauma, among others) [17].

Macular holes were considered to be untreatable before the 1991 pioneering study of Kelly and Wendel [18] who developed a surgical technique capable of closing a macular hole with good anatomical and functional results. With the technical and instrumental progression over the years, pars plana vitrectomy has become the gold standard, with posterior vitreous detachment with or without peeling of the internal limiting membrane (ILM) and gas tamponade, permitting the closure of 85–90% of the cases [19].

For macular holes measuring more than 500 µm, the final visual acuity usually is less than 0.2 and reoperations are often necessary [20, 21]. The variation in the type of postoperative macular hole closure is another factor influencing the lack of anatomical success. After primary repair by the standard technique, large macular holes remain open in up to 44% of all cases [22]. Thus, surgical alternatives are being developed for holes > 400 μm, such as ILM peeling with an inverted flap [23, 24] in order to improve the anatomical and functional results.

Therapeutic options for small and medium holes are being extensively revised. Due to the lack of randomized clinical assays with an appropriate sample, there is still no consensus for large, recurrent or persistent holes [25, 26]. For this reason, our group decided to try a different surgical technique for macular holes larger than 700 µm of minimum linear diameter (MLD), which we called very large macular holes. The current report presents a series of 4 cases subjected to the pedicle ILM flap technique with the description of anatomical and functional aspects.

## Methods

This is a retrospective series of four patients with large macular hole who were treated with pedicle ILM flap technique, between May 2018 and May 2019.

Comprehensive ophthalmologic evaluation performed at baseline and postoperative following PPV included ETDRS best-corrected visual acuity (BCVA); MH height, minimum and maximum hole diameters measured by spectral domain optical coherence tomography (OCT) (Heidelberg, Germany). According to the International Vitreomacular Traction Study (IVTS) [17], all 4 cases reported here were classified as having a large full thickness macular hole: a hole with a distance of more than 400 µm between the closest margins of the retinal gap (MLD). Indeed, all cases included had MLD higher than 700 µm and were denominated by our group as very large macular holes.

Before surgery, all eyes were dilated with two drops of 10% phenylephrine eyedrops administered 5 min apart and three drops of 1% tropicamide eyedrops administered 3 min apart. The initial treatment was provided by the same surgeon in a surgical environment under aseptic conditions and with topical anesthesia. A three-port PPV, core vitrectomy and trypan blue staining were performed. If an epiretinal membrane was present, it was peeled. The ILM was peeled in an area of 4–5 mm around the macular hole. During the peeling, the ILM was not removed completely from the retina but was left attached to the temporal edge of the macular hole. The ILM flap remains attached to the macular hole edge and is trimmed with the vitrectomy probe using the scissors mode. A pedicle ILM flap large enough to cover the entire MH diameter is left and inserted beneath the MH edges.

## Results/case series

### Case 1

**Fig. 1:**
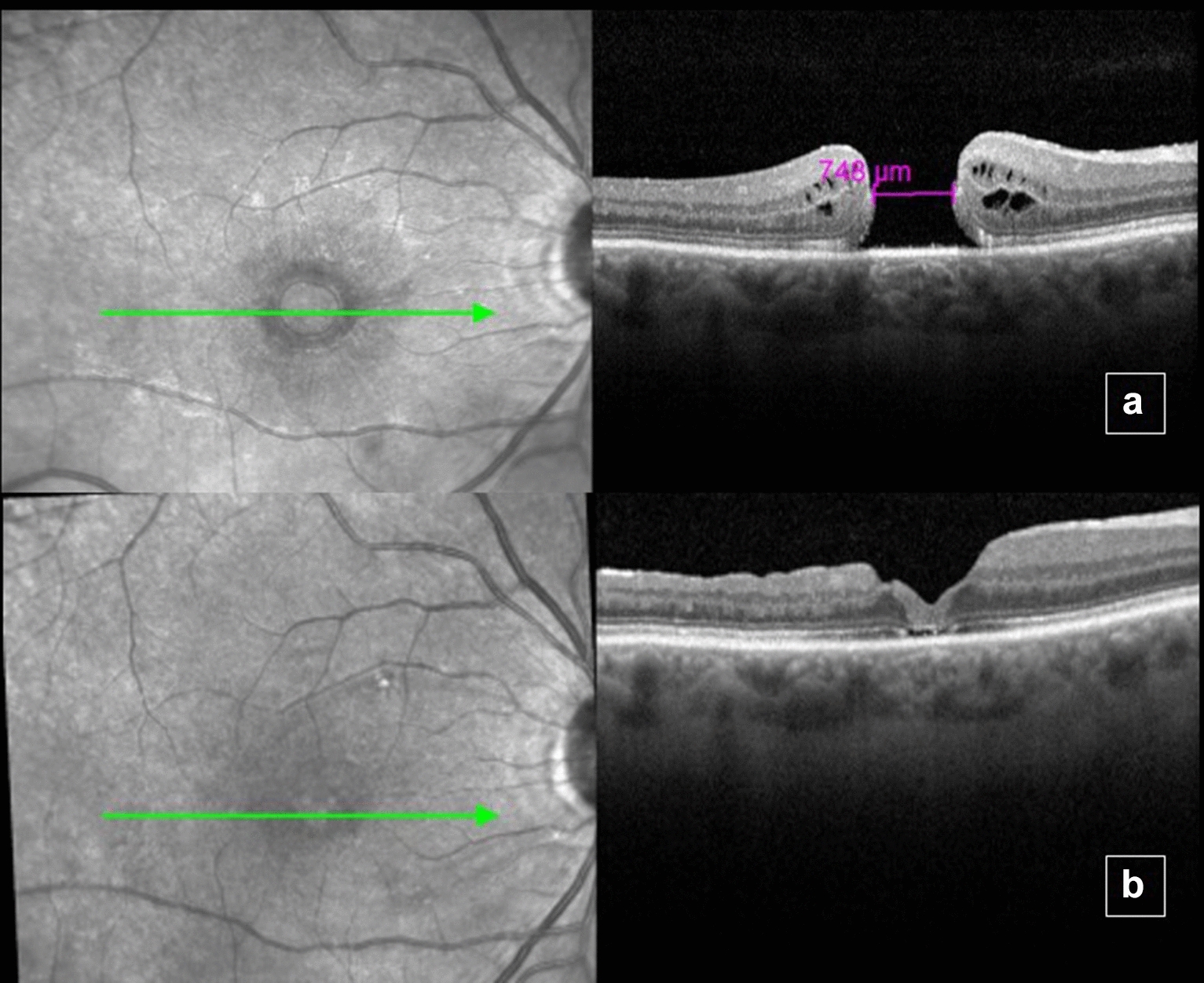
Optical coherence tomography of the right eye before surgery showing Full thickness macular hole (**a**). Five months after surgery, there was closure of the macular hole (**b**). Note the restructuring of the photoreceptor layer of the retina. In foveal center, there was partial restructuring of the external limiting membrane (ELM) layer, but no restoration of the ellipsoid zone layer

**Fig. 2:**
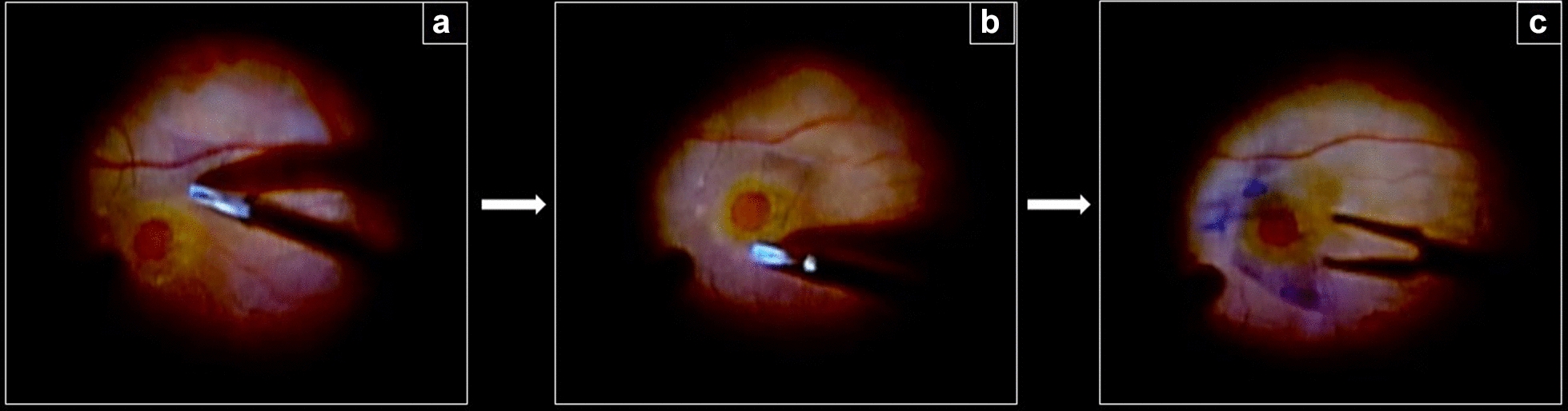
Pedicle internal limiting membrane flap technique. **a** ILM peeling starts inferiorly to the macular hole (surgeon view); **b** the ILM is carefully dissected around the macular hole; **c** The ILM flap remains attached to the temporal macular hole edge and is trimmed with the vitrectomy probe using the scissors mode

A 67-year-old woman with progressive visual loss complaint in the right eye starting approximately 8 months ago. Ophthalmological examination revealed BCVA of 1.3 logMAR (20/400 snellen) in right eye (OD) and 0 logMAR (20/20 snellen) in the left eye (OS). Patient presented preserved direct and consensual pupillary reflexes and normal applanation tonometry in both eyes. Slit lamp biomicroscopy showed topical intraocular lens (IOL) in OD. Eye fundus examination revealed a grade 4 macular hole (MH) according to Gass [4], confirmed by OCT, which showed a full thickness MH with a minimum linear diameter of 748 μm and a height of 368 μm (Fig. 1a). No changes were found in OS. The following procedures were performed: prophylactic 360° laser in OD, pars plana vitrectomy, ILM peeling with confection of an inverted pedicle ILM flap of OD followed by fluid air exchange and 13% C3F8 infusion. (Fig. 2a–c). Five months after the intervention, the patient showed BCVA of 1.0 logMAR (20/200 snellen) in OD, with full MH closure detected by fundoscopy and OCT (Fig. 1b).

### Case 2

**Fig. 3:**
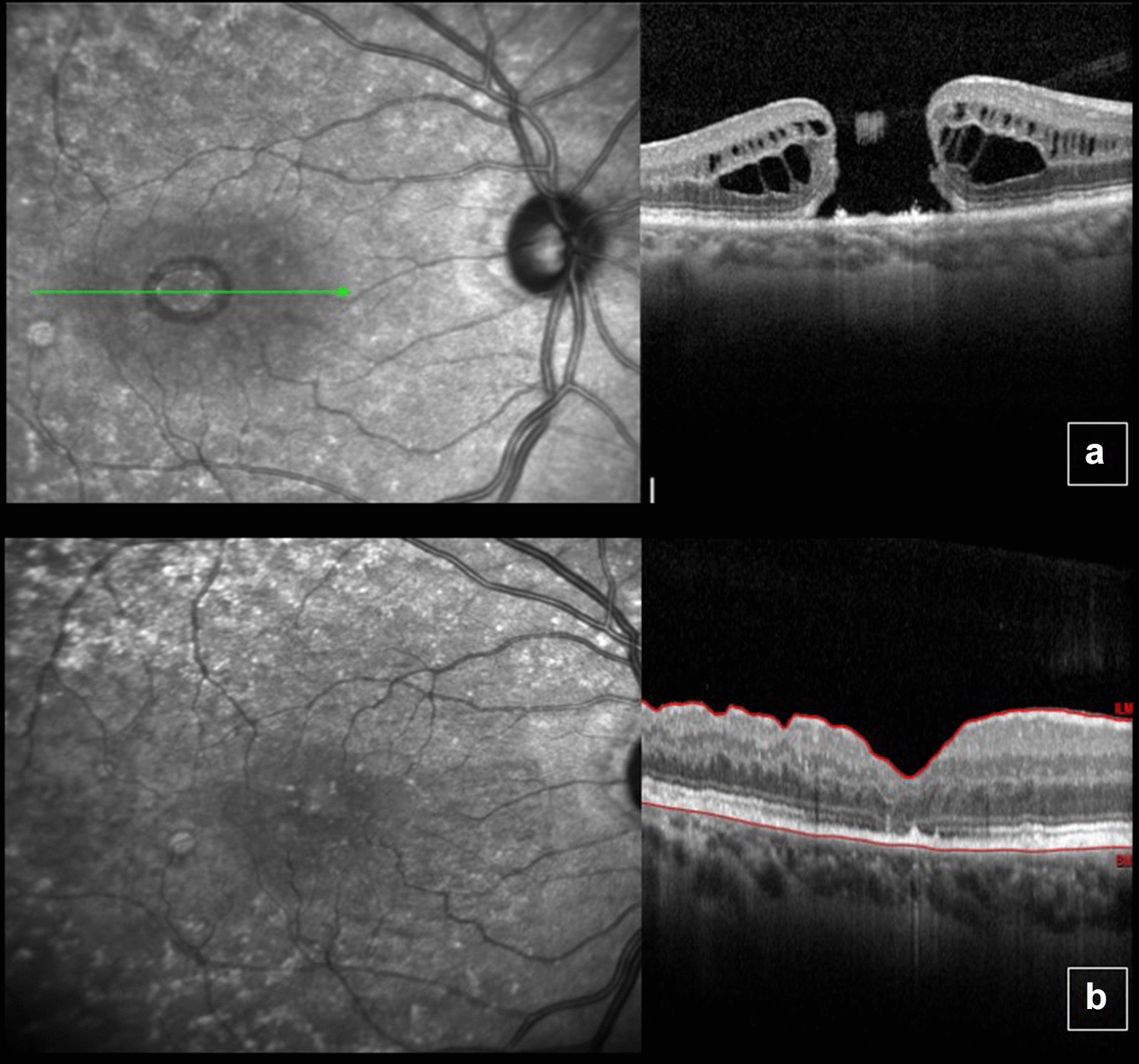
Optical coherence tomography of the right eye before surgery showing a full thickness macular hole with intraretinal cysts (**a**) and macular hole closure 6 months after surgery (**b**). Note the restructuring of the photoreceptor layer of the retina. In foveal center, there was partial restoration of the external limiting membrane (ELM) and ellipsoid zone layers

A 70-year-old woman presented progressive low visual acuity in OD for 1 year, with no previous eye surgeries. Initial examination revealed BCVA of 1.3 logMAR (20/400 snellen) in OD and 0.5 logMAR (20/63 snellen) in OS. Slit lamp examination shows moderate cataract in OD. Fundoscopy revealed a grade 4 MH in OD. OCT showed a full thickness MH with a minimum linear diameter of 811 μm and height of 444 μm (Fig. 3a). No changes in OS. The following procedures were indicated: phacoemulsification, pars plana vitrectomy and ILM peeling and confection of an inverted pedicle ILM flap in OD followed by fluid air exchange and 13% C3F8 infusion. Six months after the procedure, the patient showed BCVA of 0.9 logMAR (20/160 snellen) in OD, with fundoscopy and OCT revealing full MH closure (Fig. 3b).

### Case 3

**Fig. 4:**
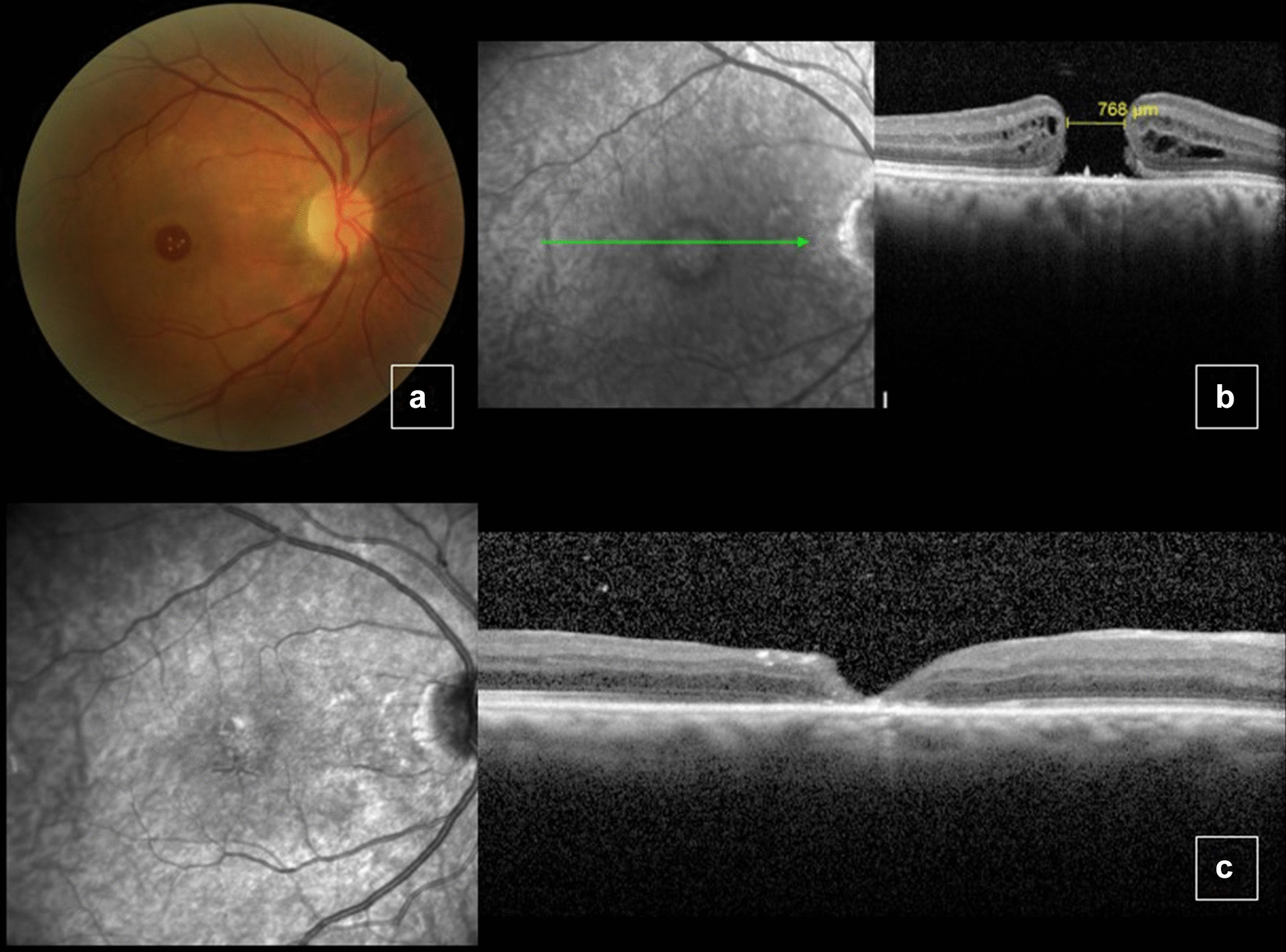
Color fundus picture and optical coherence tomography (OCT) of the right eye before surgery, showing a large macular hole (**a–b**). Five months after surgery, the macular hole was closed (**c**). In foveal center, no restoration of the external limiting membrane (ELM) and ellipsoid zone layers was verified

A 72-year-old man with presented with 5 year history of progressive visual loss in OD starting 5 years ago. BCVA was 1.3 logMAR (20/400 snellen) in OD and 0.3 logMAR (20/40 snellen) in OS. Slit lamp examination showed moderate cataract in OD. Fundoscopy revealed a grade 4 MH in OD (Fig. 4a). OCT showed a full thickness MH with a minimum linear diameter of 768 μm and a height of 388 μm (Fig. 4b). No changes in OS. The following procedure was performed: phacoemulsification, pars plana vitrectomy, ILM peeling and confection of an inverted pedicle ILM flap of OD followed by fluid air exchange and 13% C3F8 infusion. Five months after the procedure, the patient had BCVA of 1.0 logMAR (20/200 snellen) in OD, with full MH closure determined by fundoscopy and OCT (Fig. 4c).

### Case 4

**Fig. 5:**
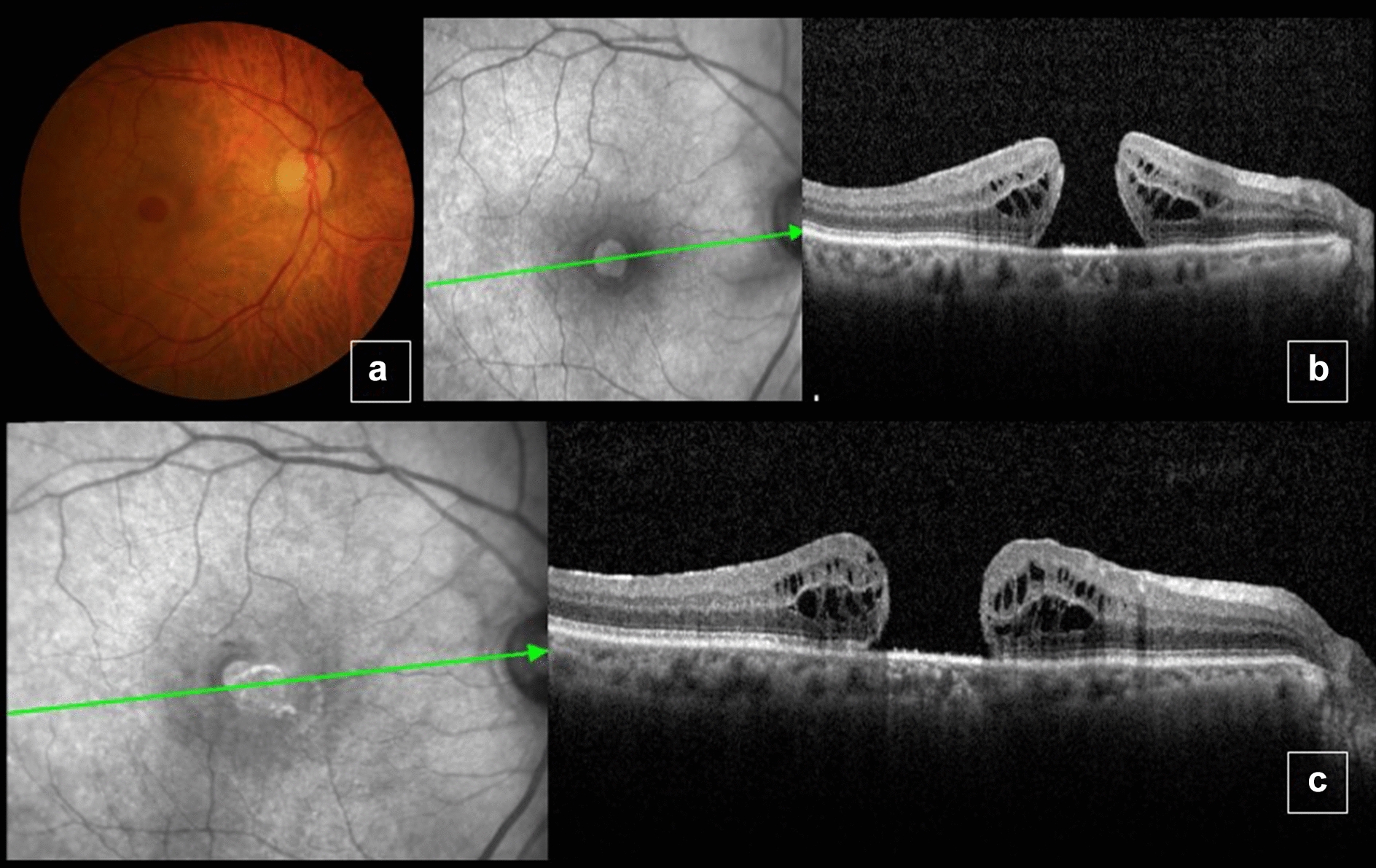
Color fundus picture and optical coherence tomography (OCT) before surgery, showing large macular hole (**a**, **b**). Six months after surgery, MH with dimensions similar to baseline values (**c**)

A 76-year-old woman with superior visual field reduction in OD starting 1 month ago. BCVA was 1.2 logMAR (20/320 snellen) in OD and 0.1 logMAR (20/25 snellen) in OS. On slit lamp examination, both eyes were phakic. Fundoscopy revealed a grade 4 MH in OD. OCT showed a full thickness MH with a minimum linear diameter of 755 μm and a height of 483 μm (Fig. 5a, b). No changes in OS. The following procedures were indicated: phacoemulsification, pars plana vitrectomy, epiretinal membrane and ILM peeling and confection of an inverted ILM pedicle flap followed by fluid air exchange and 13% C3F8 infusion**.** Five months after the procedure, the patient had maintained BCVA of 1.2 logMAR (20/320 snellen) in OD and a maintained MH of width and thickness similar to baseline values (Fig. 5c).

## Discussion

Kelly and Wendel [18] described the conventional MH surgery, but modifications have been made over the years providing better visual and anatomical results, especially for large or recurrent holes. One of these modifications was reported by Michalewska et al. [23] who used a pedicle ILM flap to close macular holes, a similar technique to the employed in our case series. We reported here patients operated with the inverted ILM pedicle flap technique for very large MH (MLD > 700 μm). Our technique, however, has some differences when compared to Michawleska`s. In her 2010 paper [23], the ILM peeling diameter was around 3 mm and the ILM was left attached to 360° of the macular edge, while in our technique, the ILM peeling diameter was larger (around 4–5 mm) and we left the ILM attached only to the temporal edge of the macular hole. Regarding Michawleska’s study from 2015 [24], there are also some differences: her group reports an ILM peeling with a 3-mm diameter and 180° temporal to the fovea. After inverting the flap and inserting it into the macular hole, they leave 180° of nasal macular ILM intact, while our technique involves a larger area of ILM peeling that also includes the nasal edge of the MH and the nasal macular region, as mentioned above.

Of the 4 cases reported, 3 obtained anatomical closure (75%). Michalewska et al. [23], in a comparative study of two groups—one of 51 eyes submitted to standard pars plana vitrectomy and the other of 50 eyes submitted to the pedicle inverted ILM flap—obtained closure of the MH in 88% of the patients in the conventional group and in 98% of the patients in the other group. Plane and open postoperative appearance was observed in 19% of group 1 patients and in 2% of group 2 patients.

Studies by Michalewska et al. [20] and Imai et al. [21] have shown that the most prevalent MH closure contour and the one related to the best functional results is the U-shaped closure, followed by the V-shaped and W-shaped closure, with the last type showing a worse functional result even in the presence of a favorable anatomical outcome. In the present study, we obtained 2 V-shaped closures and 1 U-shaped closure. In the V-shaped cases, BCVA improved one and two lines of visual acuity, respectively, while in the U-shaped closure, BCVA improved one line of visual acuity.

Another important factor for final BCVA is the continuity of the outer retina photoreceptor layer. Iwasaki et al. [27] analyzed the influence of the ILM inverted flap technique on the structures of the outer retinal layer by comparing 14 cases operated with the use of this technique to 10 cases operated with the conventional technique. The rates of postoperative recovery of the outer limiting membrane and of the ellipsoid zone in the inverted group were lower (21.4 vs. 70.0%), as also was the improvement of VA (9.0 vs. 22.5%). For this reason, the inverted flap technique should be employed just for large macular holes, and not for smaller holes that tend to close after conventional ILM peeling.

Park et al. [28], in a study comparing the inverted flap technique and the technique of ILM insertion into the MH, showed superiority of the former technique, also used in the present study, with complete resolution of the defects of the ellipsoid zone and of the outer limiting membrane being observed in 7 and 8 eyes, respectively, in the inverted flap group. In contrast, full resolution was not observed in any of the eyes of the insertion group. Mean final VA was also better in the inverted flap group.

In addition to this described technique, the options reported in the literature for large macular holes are amniotic membrane tamponade [29], autologous ILM transplant [30, 31] or neurosensory retinal free flap transplant [32]. The use of adjuvants such as autologous serum [33–35], whole blood serum [36], platelet concentrate [37, 38], thrombin [39] and tumor growth factor beta (TGF-β2) [40] has also been reported. One of the great advantages of the present technique is the use of a tissue already present in the retina, with no need of blood processing and no need for harvesting tissue from other retinal regions. In addition, the advantage of the pedicle flap is that it will not move from the edge of the macular hole during fluid air-exchange, allowing easier positioning of the ILM tissue into the hole, when compared to free flaps techniques.

The mechanism for macular hole closure using the present technique remains controversial. We agree with Michalewska et al. [23] hypothesis that the ILM flap serves as a scaffold for glial cells migration and reparative tissue formation. Besides, our technique uses a larger area of ILM peeling than the previous technique reported by Michalewska, and this may contribute to a higher release of tangential traction. Consequently, there maybe a higher chance for macular hole margins connection and subsequent closure.

On the other hand, this technique cannot be employed for persistent holes, where the ILM has already been peeled around the fovea. For this reason, our group has worked on mathematical models to predict macular closure rates using OCT parameters (Pinto et al. 2020) and use the ILM pedicle technique on the first approach for large macular holes with OCT parameters related to closure rates lower than 80%.

## Conclusion

The present study describes the first Brazilian case series of large MH treated by the inverted pedicle ILM flap technique. In macular holes > 700 μm, this technique showed preliminary promising results and further studies are warranted.

## Data Availability

The datasets used and/or analyzed during the current study are available from the corresponding author on reasonable request.

## Abbreviations

OD: Right eye
OS: Left eye
MH: Macular hole
ILM: Internal limiting membrane
BCVA: Best corrected visual acuity
SD-OCT: Spectral Domain Optical Coherence Tomography
MLD: Minimum Linear Diameter
TGF-β2: Tumor growth factor beta 2
